# The Effect of Communication on Childcare Utilization Intention Among Mothers of Children Under Three Years of Age: A Controlled Trial in China

**DOI:** 10.3390/bs16071078

**Published:** 2026-07-01

**Authors:** Hanxiao Liu, Jianghua Liu

**Affiliations:** The Institute for Population & Development Studies, School of Public Policy & Administration, Xi’an Jiaotong University, No. 28, West Xianning Road, Xi’an 710049, China; jh930.liu@xjtu.edu.cn

**Keywords:** childcare services, communication, controlled trial, theory of planned behavior, utilization intention

## Abstract

The communication of childcare services has been suggested as a major strategy to promote their development, one of the keys to counteracting the low-fertility issue; however, empirical evidence on the measure’s effectiveness remains limited. Based on the theory of planned behavior, the current study conducts a quantitative controlled policy field experiment to examine how childcare communication influences intention to utilize childcare services and its determinants among mothers with children aged 0–3 years old in Xi’an city, China (*n* = 101). The intervention included three channels of communication: banners; performances by center-cared children in the community; and online childcare classes for target mothers. Before the communication intervention, there were essentially no significant differences between the experimental and control groups in the intention-related aspects. After the intervention, the experimental group showed significant improvements in attitudes and subjective norms, while improvements in perceived behavioral control and utilization intention were not significant. To further improve the effectiveness of childcare communication, future efforts could focus on leveraging grassroots networks and expanding the target population for communication.

## 1. Introduction

In recent years, rising female labor force participation, declining fertility rates, and growing recognition of early childhood development have made childcare services a global concern (Juni García et al., 2025; Shi & Chen, 2024). Childcare services affect children’s healthy growth, early capability development, and maternal labor supply. However, the actual utilization rate remains generally low (Shi & Chen, 2026; Y. Wang & Liu, 2022). As of the end of 2023, the enrollment rate for children under three years of age in China was only 7.86% (China Youth Daily, 2024). Similarly low rates have been observed in other countries, such as Italy (26.9%, 2019), the Czech Republic (6.8%, 2017), and Poland (10.1%, 2017) (Mussino & Ortensi, 2023; Thil et al., 2023). Studies based on Chinese mothers further indicate that only 24% intend to use formal childcare services (C. Chen et al., 2023). The fundamental reason for these low utilization rates lies in parents’ low intentions.

To understand parents’ utilization intentions, scholars have conducted multidimensional investigations. Based on the theory of planned behavior (TPB) (Ajzen, 1991), we organize these factors into three categories: attitudes, subjective norms, and perceived behavioral control. Attitudes reflect parents’ rational weighing of the costs and benefits: parents are more likely to intend to use childcare when the perceived benefits outweigh the costs (Ferguson et al., 2022; Yu et al., 2025); conversely, concerns about inadequate care or doubts about developmental benefits reduce intentions (Ackert et al., 2019; Bosire et al., 2025). Subjective norms reflect the influence of family, friends, and neighbors, primarily through information and recommendations (Bosire et al., 2025; Novikova et al., 2026; Seo, 2003). Perceived behavioral control centers on parents’ perceptions of institutional quality and price. Regarding institutional quality, which encompasses physical facilities and staff qualifications, favorable perceptions (e.g., quality facilities, qualified staff, affordable prices) strengthen their perceived behavioral control, which in turn strengthens their utilization intentions (Gordon et al., 2021; Li & Liu, 2024; Novikova et al., 2026).

Although cognition plays an important role in shaping childcare demand, empirical evidence on the effectiveness of communication interventions remains relatively limited. Parents’ utilization intentions are positively correlated with their awareness of childcare services (C. Yang et al., 2025), yet most families lack sufficient knowledge (J. Chen & Liu, 2022; Davidson et al., 2022). Therefore, communication has been proposed as a tool to enhance awareness and stimulate demand (Guo et al., 2025; Li & Liu, 2024; Y. Wang & Liu, 2022). One experimental study by Galasso et al. (2017) found that providing information on childcare benefits to nulliparous women significantly increased their utilization intentions. However, that study focused on childless women and a single message type (benefits information), leaving open questions about whether similar effects would hold for mothers of young children or for messages addressing other barriers such as quality concerns. More broadly, trials in public health, social security, and other fields suggest that information interventions can change cognitions, attitudes, and behavioral intentions, but their effectiveness varies depending on message content, target population, and outcome measures (Martinez et al., 2025; Shen et al., 2025; Xiao et al., 2026). Given this variation, further research is needed to examine intervention effects in specific contexts. The present study extends prior work by: (1) targeting mothers of children aged 0–3 years; (2) designing a multi-content intervention covering childcare functions, quality information, and preferential policies; and (3) examining changes in TPB constructs and intentions.

We propose a communication intervention framework based on TPB. Regarding content, the intervention may focus on three areas: functions of childcare services (e.g., work–family balance, early development), institutional quality information, and preferential policies (Gou et al., 2024; Heinz et al., 2025). Providing factual information about childcare functions and quality can help correct misconceptions and gradually shift parents’ perceptions. Specifically, messages about childcare functions are primarily designed to target mothers’ attitudes; information on institutional quality and preferential policies primarily targets perceived behavioral control; and community-based activities (e.g., banners, performances by enrolled children) primarily target mothers’ subjective norms. Regarding communicators, given that childcare is recognized as a quasi-public good (Ren, 2023), institutional staff serve as credible messengers (Redman et al., 2022), and communities act as bridges (L. Yang, 2023), a framework led by the government, with institutions as the core and communities as the hub, could be considered (Gou et al., 2024). Regarding channels, drawing on cultural transmission theory (Cavalli-Sforza & Feldman, 1981), a combination of offline (e.g., banners, community performances) and online (e.g., WeChat, TikTok) approaches can be used to leverage horizontal peer transmission (Y. Wang & Liu, 2022).

Therefore, based on TPB, this study analyzes survey data collected from mothers of children aged 0–3 years in a western Chinese city before and after a childcare communication intervention. Specifically, we address three research questions: (1) Does the communication intervention change mothers’ attitudes, subjective norms, and perceived behavioral control? (2) Does it increase mothers’ utilization intentions? (3) Do the three TPB constructs mediate the intervention–intention relationship? Based on the empirical results, we raise some policy recommendations for promoting the development of formal childcare services in China.

## 2. Materials and Methods

### 2.1. Overview of the Study Design

We conducted a prospective controlled policy field experiment in Xi’an, Shaanxi Province, China, from 2023 to 2024, to test the effect of communication intervention on mothers’ intentions to utilize formal childcare services.

Participants were mothers of children aged 0–3 years, who were assigned to an experimental group or a control group. All participants completed a baseline survey (pretest) before the intervention. Subsequently, only the experimental group received a one-month childcare communication intervention. Half a month after the intervention ended, a follow-up survey (posttest) was conducted with both groups. Due to the nature of the intervention (e.g., banners, WeChat messages, community performances), participants may have been aware of their group assignment. However, they were unaware of the true purpose of the intervention and were not informed that a follow-up survey would be conducted. The posttest was administered without prior notice. In addition, the research team members responsible for data collection were blinded to group assignment.

Thus, despite the practical constraints that precluded randomization, this study essentially retained several core features of experimental design: an intervention group and a concurrent control group, pretest and posttest measurements, blinding of data collectors, and a standardized protocol with fidelity monitoring.

### 2.2. Participants

This study targeted mothers of childbearing age with children aged 0–3 years. Although childcare decisions often involve family negotiations, mothers remain the primary decision-makers for childcare in most Chinese families (Luo et al., 2022).

Based on the survey data (*n* = 317) from the “Factors Influencing Childcare Utilization Intentions among Mothers of Children Aged 0–3 Years” study conducted by the research team from 2023 to 2024, participants who met the eligibility criteria were included. The inclusion criteria were: (1) currently having a child aged 0–3 years; and (2) residing within the predefined study area. Half a month after the intervention ended, research team members contacted eligible respondents from the previous survey by telephone, informed them of the study’s purpose and procedures, and conducted a posttest survey with those who agreed to continue participating.

**Figure 1:**
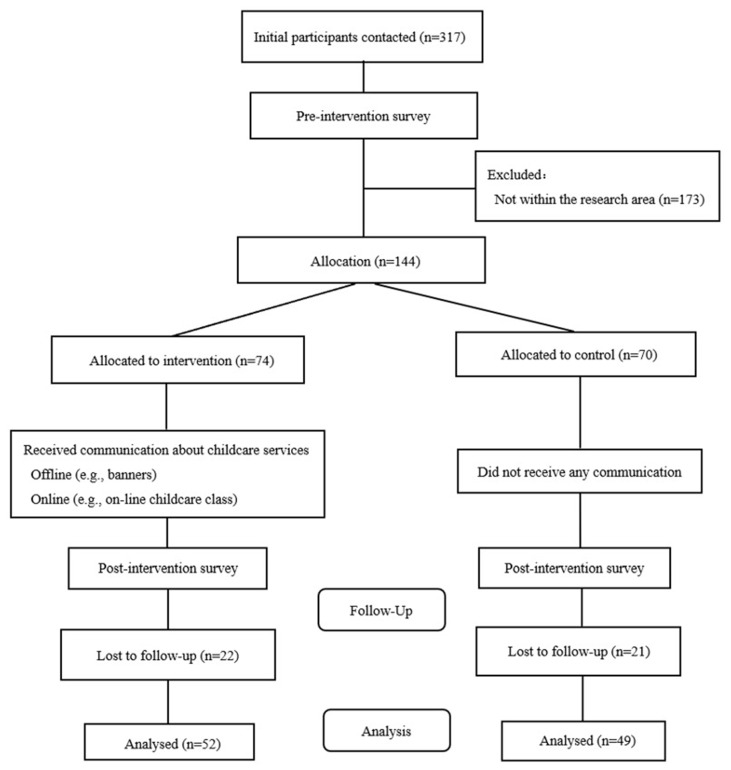
Flowchart of the controlled trial, showing participant enrollment, exclusion, allocation, intervention, follow-up, and analysis.

As shown in Figure 1, of the 317 respondents from the previous survey, 173 were excluded because their place of residence was outside the study area. The remaining 144 eligible participants were assigned to an experimental group (*n* = 74) or a control group (*n* = 70). By the time of the posttest survey, 22 participants in the experimental group and 21 in the control group were lost to follow-up, resulting in a final analytic sample of 101 participants (52 in the experimental group, 49 in the control group). The attrition rate was 29.9%.

### 2.3. Intervention

#### 2.3.1. Design

First, study sites were selected as follows. The principal investigator (PI) of the funded project used judgmental sampling to select the study sites. Taking into account administrative divisions, opinions from the municipal government, the willingness of multiple demonstration childcare institutions to cooperate, and the feasibility of intervention implementation, 5 subdistricts across 4 districts were ultimately selected as study sites (in China, a city consists of several municipal districts, and each district is divided into multiple subdistricts). Within each of the 5 subdistricts, one experimental community and one control community were designated. Group assignment was conducted at the community level and was determined by the PI based on the above factors. Only the PI was aware of the group assignment before the intervention began. Experimental communities and childcare institutions were informed of their group assignment at the start of the intervention (as they needed to implement communication activities). Control communities did not receive the intervention and were therefore not informed.

Second, intervention elements were designed as follows.

(1) Target population: Mothers who met the inclusion criteria.
(2) Intervention duration: Based on the timeframe of the government-funded project and the willingness of childcare institutions, the intervention lasted one month.
(3) Intervention communicators: Community staff and teaching staff of childcare institutions.
(4) Intervention content: This included the functions and significance of childcare services, service philosophies and advantages of institutions (e.g., physical facilities, teaching staff qualifications), and relevant preferential policies.
(5) Intervention channels: A combination of online and offline approaches was adopted, including banners, performances by center-cared children in the community, and online childcare classes.

Although randomization was not feasible in this kind of field experiment, several features of it could assure the validity of inference. First, to address any potential baseline differences between groups due to non-randomization, statistical analysis included relevant covariates and employed difference-in-differences (DID) estimation, which partly removed time-invariant group differences. Second, any potential cross-contamination between communities tended to attenuate rather than inflate the estimated intervention effect, which thus made our inference more conservative than reality.

#### 2.3.2. Implementation

The intervention process consisted of three components: building a communication platform, offline communication, and online communication, lasting a total of one month. Half a month after the intervention ended, a posttest survey was conducted. Since this study used data from the research team’s previous survey as the pretest, no additional pretest was administered. In addition, the project leader developed a standardized intervention protocol (e.g., specifying class content, etc.) and distributed it to all communities and institutions to ensure consistency of content and procedures across experimental communities.

First, a communication platform was built. A WeChat group was created in each experimental community. Community staff assisted in the process, while institutional teaching staff were responsible for forming the groups. Group members included institutional teaching staff, study participants, and the project leader. Before the intervention, after the project leader and institutional teachers confirmed the activity schedule, the teachers announced the activities in the group: (1) hanging promotional banners; (2) performances of center-cared children in the community; (3) and online childcare classes.

It should be noted that cross-contamination between communities cannot be completely ruled out, although we strongly instructed community staff to prevent such cross-contamination.

Second, offline communication was implemented. In the experimental communities, community staff collaborated with institutional teaching staff to hang banners on childcare-related themes. Slogans included “Developing High-Quality Childcare Services to Nurture the Healthy Growth of Infants and Young Children” and “Attentive Care, Reassuring Peace of Mind.” The banners remained in place throughout the entire intervention period (one month). In addition, the childcare institution organized a performance by its enrolled children in the community and distributed flyers at the same time.

Third, online communication in the form of an online childcare class was implemented. The project leader and institutional teachers predetermined the topic for the live session. Institutional teachers then posted the live session information (topic, speaker, time, viewing method, and link) via the WeChat group. The online class was held once in each community, lasting 30 to 60 min.

Fourth, intervention fidelity monitoring was performed. During the intervention period, research team members monitored implementation by visiting the communities to check banner placement and attending online classes. Although communication procedures were essentially the same for each experimental site, there was some variation in intention delivery (e.g., some communicators talked longer than others).

Finally, a posttest survey was conducted. Half a month after the intervention ended, research team members conducted the posttest questionnaire survey with participants by telephone.

### 2.4. Hypotheses

Based on TPB (Ajzen, 1991), this study proposes the following research hypotheses. This theory has been widely applied in intervention studies in fields such as health promotion and public health (Khani Jeihooni et al., 2021; Sahraei et al., 2024), and we extend it to the domain of childcare services.

First, studies in the field of health education have shown that information interventions can effectively change women’s attitudes (Cochran & Chamlin, 2005; White et al., 2015). For example, a health education intervention based on WhatsApp improved mothers’ knowledge and attitudes toward childhood fever (Altay et al., 2026); the “Breastfeeding Mom” mobile application enhanced postpartum women’s breastfeeding knowledge and attitudes (Usnawati & Hanifah, 2025). Accordingly, we propose:

**Hypothesis** **1a.** *The communication intervention has a significant positive effect on mothers’ attitudes toward the necessity of sending children aged 1–3 years to childcare.*

**Hypothesis** **1b.** *The communication intervention has a significant positive effect on mothers’ attitudes toward the perception that the benefits of sending children to childcare outweigh its drawbacks.*

Second, regarding the effects of information interventions on subjective norms, the existing findings are inconclusive. Some studies have found that interventions significantly improve subjective norms (Jeihooni et al., 2022; Zhang et al., 2009), while others have not (White et al., 2015; Williams et al., 2015). However, these studies have all focused on health management behaviors, and the effects on childcare utilization as a family decision remain unclear. In Chinese families, husbands and children’s grandparents, as core family members and direct stakeholders respectively (Liu & Lummaa, 2019; Sun et al., 2015), possess the right to participate in family education (Mao & Wang, 2023) and typically engage in family decision-making. Accordingly, we propose:

**Hypothesis** **2a.** *The communication intervention has a significant positive effect on husbands’ level of support for using childcare services.*

**Hypothesis** **2b.** *The communication intervention has a significant positive effect on child’s grandparents’ level of support for using childcare services.*

Third, evidence on the effects of information interventions on perceived behavioral control is mixed; some studies have found significant improvements (Tizvir et al., 2024; Y. Zhu et al., 2015), while others have not (Afshani et al., 2022; Williams et al., 2015). However, given that childcare decisions involve social evaluation, the modifiability of perceived behavioral control in this context is unclear. Existing studies have shown that communicating quality standards of childcare institutions and professional qualifications of teachers can enhance mothers’ recognition (Lin & Lin, 2023; Zeng et al., 2026); communicating preferential policies can alleviate economic concerns (Li & Liu, 2024; X. Zhu & Wang, 2025). Accordingly, we propose:

**Hypothesis** **3.** *The communication intervention has a significant positive effect on mothers’ perceived behavioral control regarding childcare services.*

Finally, evidence on the effects of information interventions on behavioral intention remains inconclusive. Some studies have found significant increases (Sahraei et al., 2024; Tizvir et al., 2024), while others have not (Hardeman et al., 2002; Williams et al., 2015). The modifiability of behavioral intention may depend on the type of behavior and population characteristics. Given that our study focuses on the intention to utilize childcare services, we propose:

**Hypothesis** **4.** *The communication intervention has a significant positive effect on mothers’ intention to utilize childcare services.*

### 2.5. Data Collection

Before formal data collection, the questionnaire, designed based on TPB, was piloted and reviewed by childcare experts and government officials familiar with the field for cultural appropriateness.

Study data were collected via telephone by standardized trained research team members. Participants were assured of the confidentiality of their responses and informed that there were no right or wrong answers to minimize social desirability bias. Data collection involved two phases. The first phase was the baseline survey (pretest), completed before the intervention using the questionnaire. The second phase was the follow-up survey (posttest), conducted half a month after the intervention, collecting all variables except demographic characteristics. Since the intervention lasted only one month and demographics were unlikely to change significantly, they were collected only at pretest. Upon completing each questionnaire, mothers received 30 yuan as a thank you for their telephone expenses.

The measurement indicators consisted of five parts: (1) demographic characteristics: age, education, job, annual family income, number of children of the participants’ parents and their husbands’ parents, and the child’s age; (2) attitudes: perceptions of “it is necessary to send children aged 1–3 years to childcare” and “the benefits of sending children to childcare outweigh its drawbacks”; (3) subjective norms: level of support from husbands and the child’s grandparents for using childcare services; (4) perceived behavioral control: perceptions of childcare quality, teacher professionalism, and price reasonableness; and (5) utilization intention: intention to send children to childcare institutions. Except for demographic characteristics, all variables were measured using a 5-point Likert scale. Except for institutional price, all variables were reverse-coded. For example, for attitudes, the response options ranged from “1 = strongly agree” to “5 = strongly disagree,” meaning that lower scores indicate higher agreement. For subjective norms, the response options ranged from “1 = strongly support” to “5 = strongly oppose,” meaning that lower scores indicate higher support.

### 2.6. Data Analysis

#### 2.6.1. Data Processing and Software

Questionnaire data were entered using EpiData 3.1 software, and double-checking and consistency verification were performed to ensure the accuracy of data entry. Multiple imputation in SPSS 27.0 was used to handle missing values in a very small number of predictor variables (missing rate was generally around 5%), generating 5 imputed datasets. Subsequent statistical tests and model analyses were all conducted in R 4.5.2, and the results were combined based on the small-sample adjustment method proposed by Barnard and Rubin (1999).

#### 2.6.2. Statistical Analysis Strategy

To evaluate the intervention effects, we adopted the following strategy. First, baseline balance testing was conducted. Baseline differences in demographic characteristics between groups were compared using independent samples *t*-tests (continuous variables) and chi-square tests (categorical variables). Second, descriptive statistics were calculated. Means, standard deviations, and within-group changes were calculated for each group at pretest and posttest. Cohen’s d was computed to assess the practical significance of baseline between-group differences. Third, assessment of net intervention effects was conducted. A difference-in-differences model was used to estimate these effects (see Section 2.6.3). Since participants were grouped by community, the DID model employed community-level cluster-robust standard errors. Fourth, exploratory mediation analysis was conducted. To further explore the possible mechanisms of intervention effects, and given the limited sample size and small number of communities, we conducted an exploratory mediation analysis. A parallel mediation model was used, with the intervention as the independent variable, utilization intention as the dependent variable, and attitude, subjective norm, and perceived behavioral control as parallel mediators. Because the bootstrap method does not require the assumption of multivariate normality, indirect effects were tested using bootstrap resampling (5000 repetitions) to generate 95% confidence intervals.

#### 2.6.3. Model Specification

Given the two-group, two-period design of this study (pretest and posttest for both the experimental group and the control group), the DID framework estimates intervention effects by focusing on within-group changes over time rather than absolute levels, thereby eliminating time-invariant differences between groups. To identify the net effects of the communication intervention and rule out the interference of unobservable factors, the following DID models were constructed:

Model (1): basic model (without covariates):

Y_it_ = β_0_ + β_1_Treat_i_ + β_2_Post_t_ + β_3_ (Treat_i_ × Post_t_) + ϵ_it_

Model (2): extended model (with covariates):

Y_it_ = β_0_ + β_1_Treat_i_ + β_2_Post_t_ + β_3_ (Treat_i_ × Post_t_) + γX_it_ + ϵ_it_

where the subscripts i and t represent individual participants and time (0 = pretest, 1 = posttest), respectively. Y_it_ denotes the outcome variable. Treat_i_ is the group dummy variable (1 = experimental group, 0 = control group). Post_t_ is the time dummy variable (1 = post-intervention, 0 = pre-intervention). The interaction term coefficient β_3_ represents the net effect of the communication intervention. β_0_ is the constant term. X_it_ is a vector of control variables (e.g., age, education, child’s age). Previous studies have shown that these variables are associated with childcare utilization intentions (Qiu, 2025; X. Wang et al., 2021), and they were therefore included as covariates. ϵ_it_ is the random error term.

### 2.7. Ethical Considerations

The study was conducted in accordance with the Declaration of Helsinki and approved by the Department of Social Sciences Research at Xi’an Jiaotong University (Approval No. SKH2023079; 26 April 2023). All participants were informed of the study purpose and procedures. Participation was entirely voluntary, and withdrawal at any time was permitted without consequences. The study content respected local cultural customs, and the research team had relevant professional expertise in child development and family studies.

## 3. Results

### 3.1. Demographic Characteristics of Participants

A total of 101 mothers of children aged 0–3 years were enrolled, with 52 in the experimental group and 49 in the control group. Except for child’s age, no significant differences were observed between the two groups in other demographic variables (Table 1). Participants in both groups were approximately 33 years old on average; most held a senior-college or bachelor’s degree, were employed in the public sector or state-owned enterprises and had an annual family income below 150,000 yuan. Chi-square tests showed no significant differences between the two groups for any categorical variables. Independent samples *t*-tests showed that the mean child’s age in the control group (M = 1.61, SD = 0.67) was slightly higher than that in the experimental group (M = 1.21, SD = 0.78), while no other continuous variables differed significantly. Regarding the between-group difference in child’s age, child’s age is a time-invariant individual characteristic. Therefore, in Model (2) of the subsequent regression analyses, we include child’s age as a covariate to further control for the potential influence of this baseline difference on the estimation of the intervention effect.

Sample attrition and intervention engagement. The final analytic sample consisted of 101 participants, with an overall attrition rate of 29.9% (see Figure 1). Comparison of baseline characteristics between participants who completed the study and those who were lost to follow-up revealed no significant differences except for child’s age. In addition, all 52 participants in the experimental group who completed the follow-up survey were exposed to all three intervention components, yielding an engagement rate of 100%.

### 3.2. Baseline Balance and Descriptive Statistics

#### 3.2.1. Baseline Balance Testing

The results of baseline balance testing for the core variables are presented in Table 2. After multiple comparison correction using the FDR method, no significant baseline between-group differences were found for any variable except for grandparents’ support. Grandparents’ support showed a marginally significant between-group difference at baseline (adjusted *p* = 0.059, Cohen’s d = 0.596), with a mean of 3.492 (SD = 1.158) in the experimental group and 2.845 (SD = 1.003) in the control group. Consistent with the reverse-coding rule (see Section 2.5), the experimental group actually had lower levels of grandparental support than the control group before the intervention. Regarding the marginally significant baseline difference in grandparents’ support, the DID method automatically removes time-invariant fixed differences between groups by focusing on within-group changes over time rather than on absolute levels between groups. Given that the intervention period of this study was only one month, it is unlikely that the two groups would have spontaneously developed different trends over such a short period in the absence of the intervention. Therefore, it is a relatively plausible assumption that the two groups would follow parallel trends, and this baseline difference is unlikely to confound the estimation of the intervention effect. However, since this study only includes two waves of data (pretest and posttest), the parallel trends assumption could not be statistically tested, which is a limitation of this study.

#### 3.2.2. Descriptive Statistics

The means of each variable before and after the intervention and the within-group changes are shown in Table 3. First, attitudes are considered. Regarding the attitude toward “it is necessary to send children aged 1–3 years to childcare,” the control group mean increased by 0.326, while the experimental group mean decreased by 0.058. Regarding the attitude toward “the benefits of sending children to childcare outweigh its drawbacks,” the control group mean increased by 0.368 and the experimental group mean increased by 0.019. Second, subjective norms are considered. For husband’s support, the control group mean increased by 0.351 and the experimental group mean increased by 0.211. For grandparents’ support, the control group mean increased by 0.522 and the experimental group mean increased by 0.023. Third, perceived behavioral control is considered. For perceived institutional quality, the control group mean increased by 0.261 and the experimental group mean increased by 0.131. For perceived teacher professionalism, the control group mean increased by 0.127 and the experimental group mean increased by 0.088. For perceived price, the control group mean decreased by 0.098 and the experimental group mean decreased by 0.066. Fourth, utilization intention is considered. The control group mean increased by 0.306 and the experimental group mean increased by 0.154.

### 3.3. DID Regression Results

To assess whether the baseline difference in child’s age influenced the estimated results, we included it as a covariate in Model (2). As shown in Table 4, comparing the results of Model (1) (without covariates) and Model (2) (with covariates including child’s age), the interaction coefficients for all core variables did not change substantively. This indicates that including child’s age as a covariate did not alter the estimated results, and that the baseline difference in child’s age did not meaningfully affect the study’s conclusions.

Attitudes. DID analyses showed that the communication intervention had a significant effect on mothers’ attitudes toward “the necessity of sending children aged 1–3 years to childcare,” and the results remained robust after including control variables. In Model (1), the interaction coefficient was −0.384 (SE = 0.141, 95% CI = [−0.661, −0.107], *p* < 0.01), and the results remained robust in Model (2) (β_3_ = −0.384, SE = 0.145, 95% CI = [−0.671, −0.097], *p* < 0.01). Hypothesis 1a was supported. Regarding the attitude toward “the benefits of sending children to childcare outweigh its drawbacks,” the interaction coefficient was −0.348, but it was not significant in either model (*p* > 0.05). Hypothesis 1b was not supported.

Subjective norms. The communication intervention had a significant effect on children’s grandparents’ support, but its effect on husbands’ support was not significant. For children’s grandparents’ support, the interaction coefficient in Model (1) was −0.499 (SE = 0.203, 95% CI = [−0.913, −0.085], *p* < 0.05), and the results remained robust in Model (2) (β_3_ = −0.499, SE = 0.207, 95% CI = [−0.921, −0.078], *p* < 0.05). Hypothesis 2b was supported. For husbands’ support, the coefficients in both Model (1) and Model (2) were −0.139 (*p* > 0.05). Hypothesis 2a was not supported.

Perceived behavioral control. The interaction coefficients for perceived institutional quality and teacher professionalism were negative, and the coefficient for perceived price was positive in both models, but none reached statistical significance. For perceived institutional quality, the coefficient was −0.130 in both models (*p* > 0.05). For perceived teacher professionalism, the coefficient was −0.038 in both models (*p* > 0.05). For perceived price, the coefficient was 0.033 in both models (*p* > 0.05). Hypothesis 3 was not supported.

Utilization intention. The interaction coefficients were negative in both models but did not reach statistical significance. The coefficient was −0.152 in both models (*p* > 0.05). Hypothesis 4 was not supported.

Based on Model (2), effect sizes (Cohen’s d) were calculated. The results showed that the intervention had a moderate effect on attitudes toward “the necessity of sending children aged 1–3 years to childcare” (d = 0.58). Given the low enrollment rate for children under three in China, a moderate improvement in attitudes may have practical significance. The effect size for grandparents’ support was also moderate (d = 0.53), suggesting that the communication intervention may be associated with improved grandparents’ support. Effect sizes for the remaining variables were small (d < 0.30). However, due to the limited sample size, these findings warrant replication in future studies.

### 3.4. Exploratory Mediation Analysis

To further explore the possible mechanisms underlying the intervention effects, an exploratory mediation analysis was conducted. Based on Model (2) which included demographic control variables, mediation effects were tested using bias-corrected bootstrapping with 5000 resamples, and community-level cluster-robust standard errors were employed to account for the nested structure of the data.

The results showed that the indirect effect through grandparents’ support was marginally significant at the 0.10 level (indirect effect was −0.107, 95% CI = [−0.281, 0.015]), and the indirect effect through mothers’ attitudes toward the necessity of sending children aged 1–3 years to childcare also approached marginal significance (indirect effect was −0.063, 95% CI = [−0.174, 0.015]). The indirect effects for the remaining five mediating variables did not reach significance. The direct and total effects of the intervention on the outcome were 0.182 (95% CI = [−0.132, 0.496]) and −0.152 (95% CI = [−0.444, 0.140]), respectively.

## 4. Discussion

Based on the results of a controlled trial, this study found that the childcare communication intervention was associated with improvements in some, but not all, of the theory of planned behavior constructs among mothers of children aged 0–3 years. Specifically, the intervention significantly improved mothers’ attitudes toward “the necessity of sending children aged 1–3 years to childcare” and subjective norms (primarily reflected in grandparents’ support). However, improvements in perceived behavioral control and utilization intention did not reach statistical significance. Exploratory mediation analysis showed that the indirect effect through grandparents’ support approached marginal significance, while the indirect effects of the other variables were not significant. These findings provide preliminary empirical evidence for understanding the complex mechanisms through which communication interventions influence childcare utilization intention and point to directions for optimizing childcare communication strategies.

Unlike some previous studies that remained at the level of policy recommendations or theoretical discussions (J. Chen & Liu, 2022; Li & Liu, 2024; C. Yang et al., 2025), the present study employed a controlled trial design and provided preliminary empirical evidence for the association between childcare communication interventions and improvements in mothers’ TPB-related constructs. The findings showed that the intervention significantly improved some dimensions of mothers’ attitudes and subjective norms regarding childcare services. Exploratory mediation analysis further revealed that the indirect effect through grandparents’ support on utilization intention approached marginal significance. It can be tentatively concluded that childcare communication has begun to show preliminary effectiveness in improving certain psychological constructs. Although this study was based on a survey of mothers of children aged 0–3 years in China, work–family conflict, childcare difficulties, and early childhood development are common issues faced by mothers globally (Cowling & Van Gordon, 2022; Nomaguchi & Fettro, 2019; Sztányi-Szekér et al., 2024). Mothers in many countries also face problems such as low awareness and recognition of childcare services and lack of clarity regarding policy content (Cook et al., 2025; Davidson et al., 2022; Heinz et al., 2025). Therefore, this communication-based approach may have some relevance for countries and regions where childcare services are still emerging, although cultural and policy differences across contexts should be carefully considered.

It should be noted that despite significant improvements in attitudes and subjective norms, utilization intention did not increase correspondingly. Several factors may explain this apparent inconsistency. According to the theory of planned behavior, attitudes, subjective norms, and perceived behavioral control jointly influence behavioral intention. In this study, perceived behavioral control did not show significant improvement, which may have weakened the facilitating effects of attitudes and subjective norms on intention. In other words, even if mothers become more cognitively convinced of the benefits of childcare and perceive greater family support, if they still worry about service quality, teacher qualifications, or affordability, their utilization intention may remain low. Furthermore, it is necessary to distinguish between improving perceptions of childcare and influencing actual childcare decision-making behavior. Actual childcare decisions are also constrained by broader structural and economic factors, such as service accessibility and cost, which a brief communication intervention cannot fully address. At the same time, translating improved attitudes into intentions may require a longer intervention period, and the single-item measure may not have fully captured subtle changes in intention; social desirability bias may also have had some influence. These findings suggest that childcare communication interventions should address all three TPB constructs, particularly perceived behavioral control.

Based on the findings, preliminary recommendations are proposed from two aspects: communicators and target population. First, it is advisable to further leverage grassroots administrative staff. The community-level implementation experience of this study indicates that subdistrict offices and communities maintain close contact with families of young children and can assist childcare institutions in conducting communication activities. The generalizability of this mode needs to be explored based on local conditions. In addition, future research could combine structural support measures such as service availability and price subsidies to comprehensively evaluate the synergistic effects of communication interventions and structural policies on childcare decision-making. Second, it is advisable to expand the target population. Given that husbands are also decision-makers in family childcare arrangements, similar communication efforts may have utility for this population. From a TPB perspective, adapting communication content to address fathers’ beliefs and normative referents could likewise influence their childcare-related intentions.

Despite the above findings, there could be a series of limitations with our study. First, our communication intervention did not follow a completely randomized experiment lasting for sufficient time. Thus, assignment of experimental and controlled groups might involve some selection bias and with only two waves of data, we cannot evaluate the parallel between them. Second, the sample size was limited and the number of communities was small, which may have limited power of statistical inference. For example, due to this limitation, the findings from exploratory mediation analysis should be taken only as preliminary clues of the effect of communication, despite support from bootstrapping sampling. Third, there could be limitations with the validity of measurements. Most TPB-related variables were assessed using only single- or two-item measures, which should be interpreted as conceptually relevant proxy indicators rather than fully validated construct scales. Additionally, the study partially relied on self-reported data from mothers (i.e., questionnaire answers may be still subject to subjective desirability bias, despite our objective validity check) and did not track actual childcare enrollment behavior. Given these limitations, the findings from our prospective controlled policy field experiment can be only taken as preliminary conclusions and warrant further examination.

## 5. Conclusions

Based on a controlled trial of mothers of children aged 0–3 years in western China, this study provides preliminary empirical evidence for the effectiveness of childcare communication interventions. Through three channels (banners, community performances by enrolled children, and online childcare classes), the intervention significantly improved mothers’ attitudes toward the necessity of sending children aged 1–3 years to childcare and subjective norms, primarily reflected in grandparents’ support. However, improvements in perceived behavioral control and utilization intention did not reach statistical significance. Exploratory mediation analysis showed that the indirect effect through grandparents’ support approached marginal significance. These findings suggest that communication interventions may positively influence certain psychological constructs, although their effect on utilization intention did not reach statistical significance in the current study.

On this basis, government sectors could consider exploring pilot programs for sustained childcare communication in suitable areas, providing policy support and financial subsidies to encourage collaboration between communities and childcare institutions. Childcare institutions, under government coordination, could implement communication activities and deliver accessible childcare information to target families. Future efforts could expand target populations, especially by taking fathers into account.

## Figures and Tables

**Table 1:** Demographic characteristics and baseline comparisons of participants.

Variables	Control Group (*n* = 49)	Experimental Group (*n* = 52)	t/χ^2^	*p*
Age	33.10 (4.22)	33.75 (3.52)	−0.84	0.403
Education			0.254	0.881
Primary school or middle school or vocational middle school	4 (8.16)	3 (5.77)		
Senior-college or bachelor’s degree	38 (77.55)	42 (80.77)		
Master or higher degree	7 (14.29)	7 (13.46)		
Job			0.458	0.928
Public sector or state-owned enterprise	21 (42.86)	23 (44.23)		
Private company or small business or joint venture or foreign-invested enterprise	13 (26.53)	15 (28.85)		
Other	10(20.41)	8 (15.38)		
Unemployed	5 (10.20)	6 (11.54)		
Annual family income			2.084	0.720
Less than 50,000 ¥	4 (8.16)	6 (11.54)		
50,000–100,000 ¥	10 (20.41)	16 (30.77)		
100,000–150,000 ¥	13 (26.53)	11 (21.15)		
150,000–200,000 ¥	7 (14.29)	6 (11.54)		
More than 200,000 ¥	15 (30.61)	13 (25.00)		
Number of children of the participant’s parents	2.04 (0.84)	1.88 (0.78)	0.967	0.336
Number of children of the husband’s parents	1.73 (0.76)	1.83 (0.88)	−0.563	0.575
Child’s age	1.61 (0.67)	1.21 (0.78)	2.691	0.008

Note: Continuous variables are presented as mean (SD); categorical variables are presented as n (%).

**Table 2:** Baseline balance testing of core variables.

Variables	Baseline Mean Difference	Cohen’s d	Adjusted *p*
Attitude 1: It is necessary to send children aged 1–3 years to childcare.	0.296	0.311	0.323
Attitude 2: The benefits of sending children to childcare outweigh its drawbacks.	0.265	0.254	0.323
SN 1: Husband’s level of support for using childcare services.	0.258	0.231	0.323
SN 2: Child’s grandparents’ level of support for using childcare services.	0.647	0.596	0.059
PBC 1: Perception of childcare institution quality.	0.240	0.328	0.323
PBC 2: Perception of childcare teachers’ professionalism.	0.165	0.227	0.323
PBC 3: Perception of the reasonableness of childcare prices.	−0.112	−0.148	0.544
Intention: The intention to send children to childcare institutions.	0.287	0.234	0.323

Note: Baseline mean difference = experimental group mean − control group mean; *p*-values were corrected using the FDR method.

**Table 3:** Pre- and post-intervention means and changes in variables by group.

Variables	Control Group (*n* = 49)			Experimental Group (*n* = 52)		
	Pretest	Posttest	Change	Pretest	Posttest	Change
Attitude 1	2.531 (0.915)	2.857 (1.041)	0.326	2.827 (0.985)	2.769 (0.783)	−0.058
Attitude 2	2.408 (1.098)	2.776 (1.085)	0.368	2.673 (0.985)	2.692 (0.829)	0.019
SN 1	2.853 (0.988)	3.204 (0.979)	0.351	3.112 (1.232)	3.323 (1.173)	0.211
SN 2	2.845 (1.003)	3.367 (1.055)	0.522	3.492 (1.158)	3.515 (1.205)	0.023
PBC 1	2.780 (0.652)	3.041 (0.789)	0.261	3.019 (0.798)	3.150 (0.754)	0.131
PBC 2	2.665 (0.743)	2.792 (0.832)	0.127	2.831 (0.715)	2.919 (0.883)	0.088
PBC 3	2.343 (0.743)	2.245 (0.715)	−0.098	2.231 (0.766)	2.165 (0.740)	−0.066
Intention	2.694 (1.228)	3.000 (1.354)	0.306	2.981 (1.229)	3.135 (1.172)	0.154

Note: Data are presented as mean (SD). Change = posttest − pretest.

**Table 4:** DID regression results.

Variables	Model	β	SE	t	*p*	95% CI
Attitude 1	(1)	−0.384	0.141	−2.734	0.007 **	[−0.661, −0.107]
	(2)	−0.384	0.145	−2.643	0.009 **	[−0.671, −0.097]
Attitude 2	(1)	−0.348	0.247	−1.409	0.160	[−0.835, 0.139]
	(2)	−0.348	0.256	−1.362	0.175	[−0.852, 0.156]
SN 1	(1)	−0.139	0.136	−1.028	0.309	[−0.412, 0.133]
	(2)	−0.139	0.139	−1.004	0.320	[−0.419, 0.140]
SN 2	(1)	−0.499	0.203	−2.458	0.020 *	[−0.913, −0.085]
	(2)	−0.499	0.207	−2.411	0.022 *	[−0.921, −0.078]
PBC 1	(1)	−0.130	0.231	−0.566	0.572	[−0.586, 0.325]
	(2)	−0.130	0.238	−0.549	0.584	[−0.601, 0.340]
PBC 2	(1)	−0.038	0.195	−0.195	0.846	[−0.428, 0.352]
	(2)	−0.038	0.200	−0.190	0.850	[−0.438, 0.362]
PBC 3	(1)	0.033	0.256	0.127	0.899	[−0.485, 0.550]
	(2)	0.033	0.261	0.125	0.901	[−0.496, 0.561]
Intention	(1)	−0.152	0.144	−1.056	0.292	[−0.437, 0.132]
	(2)	−0.152	0.149	−1.021	0.309	[−0.447, 0.142]

Note: ** *p* < 0.01, * *p* < 0.05. Model (1): without covariates; Model (2): with demographic covariates.

## Data Availability

The data presented in this study are available on request from the corresponding author due to participant confidentiality.
